# Mapping the theories, content, and outcomes of family-based interventions for children and young people with gaming disorder: A scoping review protocol

**DOI:** 10.12688/f1000research.134800.1

**Published:** 2023-09-20

**Authors:** Harshini V S, Mariam Abraham, Samyuktha Prabhu, Jing Shi, Deena Dimple Dsouza

**Affiliations:** 1 Department ofOccupational Therapy, Manipal College of Health Professions, Manipal Academy of Higher Education, Manipal, Karnataka, India; 2 Occupational Therapy Programme, Health and Social Sciences, Singapore Institute of Technology, Singapore, Singapore

**Keywords:** Gaming Disorder, Family-Based Interventions, family, scoping review, video gaming, treatment

## Abstract

**Background**

Despite the growth of gaming disorders globally, evidence of the formal involvement of family in treating gaming disorders is limited. When children are affected by gaming disorder, the family may encounter challenges in managing the behavior and in the lack of information regarding the gaming disorder, resulting in inconsistent parenting, which may further exacerbate the problem. Thus, it is essential to involve the family in formal interventions. The current scoping review plans to identify the theories, content, and outcomes of family-based interventions for children and young people with gaming disorders.

**Methods**

This scoping review will follow the Joanna Briggs Institute (JBI) methodology. The population, Concept, and Context (PCC) were used to develop the review question. The studies published in the indexed databases will be searched systematically, and the reference list of included full texts will be searched for relevant studies. Intervention studies published in English from January 2010 to December 2022 will be included. Two independent reviewers will screen the studies against eligibility criteria. The data will be extracted and presented in a tabular and narrative style.

**Discussion**

This scoping review will help better understand content, outcomes, and theories underpinning family-based interventions for children and young people with gaming disorders. Findings will inform the stakeholders about the current topic and guide the potential research areas.

**Registration details:** The protocol has been registered in Open Science Framework with the DOI: https://doi.org/10.17605/OSF.IO/TXWBH.

## Introduction

Online gaming is a popular leisure activity in the present digital age. The highly evolving process includes no gaming to occasional gaming, high-frequency gaming, and uncontrolled gaming. Online gaming has attracted an increasing number of children and adolescents ^1^ i. Furthermore, its availability 24/7 makes online gaming possible to engage children and adolescents any time of the day and for a prolonged period. Undoubtedly, the Coronavirus disease 2019 (COVID-19) pandemic has caused increased numbers in gaming disorders ^2^.

Gaming may become problematic if it causes disruptions in school and work life ^3, 4^, sleep ^5– 7 ^and social life ^5^. Dysfunctional gaming patterns have become a significant concern for children and adolescents in recent years, with increasing research publications since the World Health Organization’s inclusion of gaming disorders in the International Classification of Diseases 11 ^th^ edition ^8^. Problematic gaming behavior may not be connected only to intra-personal characteristics of an individual but also involve social variables, including parental and family factors ^9^.

The family is the primary unit for social skills development for children, and the parent-child relationship remains vital. Families assist in organizing a child’s daily needs, and a supportive family offers the most reliable network throughout childhood, early adulthood, and beyond. The family develops a special emotional bond with their child through nurturing ^10^. From a clinical perspective, including families in intervention is critical as parents may help regulate the gaming behavior of children and young people, as positive parenting and family dynamics were associated with decreased rates of problematic gaming while positive attachment with parents was associated with lower rates of problematic gaming ^9^. However, an authoritative or demanding parenting style correlated with higher rates of problematic gaming ^9^. In addition, the dysfunctional family relationship also contributed to an increase in gaming behavior ^11^.

A dearth of literature exists on interventions to prevent or treat gaming disorders. The current interventions available for gaming disorders include cognitive behavioural therapy, family therapy, motivational interviewing, individual counselling, solution-focused therapy, and combinations of these ^12^. Despite the growing gaming behavior among children and young people, there is limited research on family-based interventions. Including parents in the intervention promotes children’s initial engagement and ongoing participation. It also helps the parents learn to set limits on playing video games and (re)establish communication with children ^13^. To the best of our data search, no previous reviews have been carried out to methodically map and categorize family-based interventions for children and young people with gaming disorders. Therefore, this review is aimed to map the theories, content, and outcome of family-based interventions.

### Review question

This scoping review aims to map the theories, content, and outcome of family-based interventions for children and young people with gaming disorders.

1. What are the theories, content, and outcomes presented across interventions for families with children and young people with gaming disorders?
2. Which interdisciplinary health care professionals ( *e.g.*, educational background, qualifications) are involved in family-based interventions for children and young people with gaming disorders?
3. What functional outcomes are reported regarding family-based interventions with children and young people with gaming disorders? 

## Protocol

This scoping review is developed following Joanna Briggs Institute (JBI) Manual for scoping review ^14^. The results will be reported according to the Preferred Reporting Items for Systematic Reviews and the Meta-Analyses extension for Scoping Reviews (PRISMA-ScR). The Population, Concept, and Context (PCC) were used to develop the review question. JBI manual for scoping review guides the inclusion of studies that will be included in this review. A pilot search of PubMed and the Cochrane Database of Systematic Reviews was conducted, and no reviews were identified. If there are any changes to the protocol, the final review will amend with justification. The primary outcome of this review is to identify the theories, content and outcome of family-based interventions for children and young people. The secondary outcome health professionals involved and the functional outcome. The scoping review protocol is registered on 23 ^rd^ April 2023 in Open Science Framework (Registration DOI: https://doi.org/10.17605/OSF.IO/TXWBH) ^15^.

### Eligibility criteria

***Participants*.** This scoping review will consider the studies investigating family-based interventions to prevent or treat gaming disorders between parents of children and young people, primary caregivers, and professionals (psychiatrists, nursing, occupational therapist, and schoolteachers). The age range selected for children is from (0–10 years) and young people from (10–24 years), a term used by World Health Organization to combine adolescence and youth ^16^. Studies with participants receiving unimodal or multi-model family-based interventions will also be included. Studies with children and young people with co-occurring conditions will also be considered. 

***Concept*.** The concept of interest is any research addressing family-based interventions to prevent or treat gaming disorders for children and young people. The review will consider family defined as parents, primary caregivers such as grandparents who live with the child, and legal guardians. The studies included in this review must explicitly discuss family-based interventions for gaming disorders. In addition, the program development and validation of family-based interventions will be considered. 

***Context*.** This scoping review will consider studies involving family-based interventions for children and young people with gaming disorders conducted in physical contexts such as schools, hospitals, private clinics, outpatients, community, or digital platforms. The studies eligible for review will not be limited to any geographical location. 

***Types of evidence sources*.** This scoping review will consider all interventional studies, such as randomized controlled trials, pre-test-post-test study designs, non-randomized controlled trials, quasi-experiments, pilot studies, case series, case reports, and prospective and retrospective clinical studies. In addition, reviews and mixed-method studies will be included. Qualitative studies focusing on developing and evaluating the effects of interventions will also be eligible. The following studies will be excluded: studies that are not written in English, conference presentations, abstracts, theses, studies with participants older than 24 years old and studies published before 2010.

### Search strategy

Studies published in indexed databases PubMed, Scopus, EMBASE, and CINAHL will be searched. An initial search was developed and trailed in PubMed, Scopus, and CINAHL. Subsequently, the most suitable keywords and indexed terms were identified, and a complete search strategy was developed and trailed in PubMed, Scopus, and CINAHL. The strategy was adapted for each search since the index terms varied in some databases ( Table 1). A librarian was consulted with search terms, words, and databases. Reference lists of included studies will be hand searched to identify other relevant studies. The studies published in the years 2010–2022 will be included in the review. An extensive study range was considered, as no previous reviews were published on family-based interventions for children and young people with gaming disorders.

**Table 1.** Search strategy.

Scopus, PubMed, CINAHL, EMBASE	
Participants	“Digital gam *” OR “computer gam *” OR “video gam *” OR “online gam *" OR “gaming addiction” or “mobile gam *” OR “Internet gam *” OR “compulsive gaming” OR “excessive gaming” OR “gaming addiction" OR “gaming dependency” OR “gaming disorder” OR “pathological computer gaming” OR “pathological gaming” OR “pathological internet gaming” OR “pathological video gaming” OR “problematic computer gaming” OR “problematic digital gaming” OR “problematic gaming” OR “problematic internet gaming” OR “problematic mobile gaming” OR “problematic online gaming” OR “problematic video gaming” OR “problematic videogaming” OR “video game addiction” OR “videogame addiction”
Intervention	“Family based therap *” OR “family centered intervent *” OR “family focused therap *” OR “caregiver educat *” OR “parental educat *” OR “parenting strategies” OR “family care” OR “psychotherap *” OR Family OR parent OR “family therapy” OR psychoeducation

*MeSH headings will be used in PubMed when appropriate

### Study selection

The studies will be collated and exported to Microsoft Excel for Microsoft 365 with citation details and abstract keywords from all the electronically searched databases. Subsequently, duplicates will be removed. The two independent reviewers will screen selected sources' titles and abstracts to identify the eligibility criteria for full-text extraction. The reason for exclusion will be recorded in the scoping review presented in a PRISMA-ScR. A third reviewer will be consulted for any conflicts. The final results of the search will be reported in the scoping review and represented in the PRISMA-ScR flow diagram ^17^.

### Data extraction

Two independent reviewers will extract the data, and the third reviewer will be consulted on any discrepancies and perform a final check. The data to be extracted is outlined in Table 2 with the basic summary of the studies. The main interest of the review is presented in Table 3.

### Data extraction chart

**Table 2.** Basic summary of the eligible studies.

Publication title, author and year
Study year, location, setting, study design
Health professionals
Measures used
Outcome measures post-intervention at each stage of follow‐up

**Table 3.** Mapping of theories content, the outcome of a family-based intervention.

Author Title Publication date	Theoretical underpinning Practice approach (Frame of Reference [FOR], theories, guiding principles, and models)	Service delivery -experimental group (Intervention details type and frequency, delivery mode, session duration, duration follow-up)	Service delivery -Control group (Intervention details type and frequency, delivery mode, session duration, duration follow- up)	Outcomes	Functional outcomes

### Data analysis and presentation of the results

A summary table will be provided with detailed information on every included source as per the predefined data extraction sheet. We will summarize the first objective by grouping the theories used in family-based interventions along with content of the development program and what was the outcome of the intervention. The second objective will list health care professionals involved in the intervention. The third objective will report the functional outcome of the intervention. Descriptive analysis will include frequencies of theories used and outcomes. Furthermore, the findings will be described in a narrative style, organized according to the theories underpinning the family-based interventions.

## Ethics and dissemination

We will conduct the scoping review of existing studies and will not recruit any participants directly; thus, ethical approval will not be required. We intend to submit the paper for publication in a peer-reviewed journal. 

## Study status

We have developed the keywords for the search strategy and will run the search in predefined databases by the end of August 2023.

## Discussion

Family-based interventions' underpinning theories and content with children and young people with gaming disorders are relatively new. A scoping review will be the best method as it will ensure the literature covered is as broad as possible from all the multidisciplinary studies. It is anticipated that the results of this review will provide clear and in-depth evidence of the theories applied in family-based interventions. Publishing a planned scoping review protocol is in keeping the good transparent research practice. The synthesized knowledge will describe family-based interventions and the theories, content, and outcomes they entail. This review will help inform various stakeholders, including researchers, clinicians, and therapists from various disciplines, on the importance and applications of family-based interventions. The outcome of the scoping review will also inform future research planning. For example, researchers can collaborate with the existing family-based interventions that various healthcare professionals, organizations and schools implement, and evaluate them in different locations and cultures. The limitations expected in the scoping review will be only studies published in English will be included, studies with important data is missed if published in other languages.

## Data Availability

No data associated with this article. OSF: Mapping the theories, content, and outcomes of family-based interventions for children and young people with gaming disorder: A Scoping Review Protocol. https://doi.org/10.17605/OSF.IO/TXWBH ^15^ OSF: PRISMA-P and PRISMA-ScR checklist for “Mapping the theories, content, and outcomes of family-based interventions for children and young people with gaming disorder: A Scoping Review Protocol” https://doi.org/10.17605/OSF.IO/TXWBH ^15^ Data are available under the terms of the Creative Commons Attribution 4.0 International license (CC-BY 4.0).
