# Supplementary material for: Mapping the theories, content, and outcomes of family-based interventions for children and young people with gaming disorder: A scoping review protocol
Source: F1000Res. 2023 Sep 20;12:1178. [Version 1] doi: 10.12688/f1000research.134800.1 (PMC10924826; doi:10.12688/f1000research.134800.1)
Supplement: Peer review history

# Reviewer report: Reviewer response for version 1

**DOI:** 10.5256/f1000research.147883.r252048

**Published:** 2024-03-09

**Authors:** Marcos Bella-Fernández

**Affiliations:** 1 Hospital Universitario Puerta de Hierro Majadahonda, Majadahonda, Madrid, Spain; 2 Universidad Autonoma de Madrid (Ringgold ID: 16722), Madrid, Community of Madrid, Spain

This article describes a protocol for a scoping review which the authors are intended to perform on family-based interventions in gaming disorders in young patients (children, adolescents, and young adults). Family-based interventions have been designed to treat gaming disorders on the basis that family and social variables may impact its appearance and treatment.

 Abstract

 The abstract clearly summarizes the goal and the rationale of this scoping review. It also emphasizes that the protocol is registered in OSF. No improvement is needed.

 Introduction

 The background states that gaming disorder is a prevalent disorder among young people. Research on gaming disorder is growing to the point that it was included in the ICD-11 as a disorder. I would add that it was also recently mentioned in the DSM-5-TR under the name of Internet Gaming Disorder.

 The introduction focuses on therapies and specifically on family-based interventions. They also argue that family and social variables have an impact on gaming disorder appearance; here lies the rationale for this type of interventions.

 Review question

 The authors pose three review questions, regarding the theories, content, and outcomes of these interventions, which professionals administer them, and the reported functional outcomes. The questions are scientifically sound and easily operationalized. I have no comments on them.

 Protocol

 The authors will follow the Joanna Briggs Institute Manuslfor scoping review. I am not familiar with this, so perhaps the authors could briefly say what characterizes this manual, if that is possible. They will elaborate a report following the PRISMA-ScR guidelines.

 There is one sentence in the protocol paragraph which appears to have something missing: "The secondary outcome health professionals involved and the functional outcome". Maybe it should be something like: "The secondary outcome *is *health professionals involved and the functional outcome"?

 The authors used the Population, Concept, and Context approach to develop the research questions. As mentioned earlier, the questions are perfectly developed in my opinion.

 Regarding the sources, the authors are in general including peer-reviewed articles and excluding these and other grey literature sources. Also, the syntaxes described in Table 1 appear to we exhaustive enough to capture many valuable entries, so perhaps grey literature is not necessary in this review. However, if the authors see that they have not retrieved a sufficient number of entries, perhaps they will have to contemplate including these and other grey literature. I would also suggest the authors to include searches in PsycInfo, apart from the many other databases they are already going to use.

 The plan to present and disseminate the results seems perfectly valid for me. The only issue is the date for the research; perhaps it should be updated if the authors have not performed the searches in August 2023.

 In summary, this scoping review promises to be a valuable addition to the existing literature. Best of luck with it.

Is the study design appropriate for the research question?

Yes

Is the rationale for, and objectives of, the study clearly described?

Yes

Are sufficient details of the methods provided to allow replication by others?

Yes

Are the datasets clearly presented in a useable and accessible format?

Not applicable

Reviewer Expertise:

Literature reviews, video games, gaming disorder.

I confirm that I have read this submission and believe that I have an appropriate level of expertise to confirm that it is of an acceptable scientific standard.

# Reviewer report: Reviewer response for version 1

**DOI:** 10.5256/f1000research.147883.r233826

**Published:** 2024-02-07

**Authors:** Ben J Riley

**Affiliations:** 1 College of Medicine and Public Health, Flinders University, Adelaide, South Australia, Australia

This article reports a priori protocol for a scoping review. The scoping review intends to review the theories, content, and outcomes of family-based interventions for children and young people with gaming disorder.

 Abstract

 The abstract is clear and provides all the necessary information.

 Introduction

 The term online gaming is introduced. Gaming can of course occur both on and offline. The opening paragraph suggests this scoping review will focus on online gaming. If this is correct, I suggest an explanation as to why online gaming is the focus along with a distinction between on and offline gaming (gaming disorder naturally can include both on and offline gaming). If the focus is not intended to be online gaming only, I suggest removing the first word “Online” and simply opening with “gaming”.  

 The negative effects of excessive gaming are often sensationalised by the media. Gaming indeed increased during COVID-19, however, this was also associated with many benefits for people (as several articles report). Gaming, as we know, has many benefits for young and older people. I like to see a sentence or two noting such benefits in the introductions of articles that address the harms associated with gaming. E.g., “While there are several positive effects of engaging in gaming such as [creativity, cognition, social, physical…] excessive gaming can be harmful….”

 The justification for the scoping review is sound.

 The three review questions are clear and measurable.

 Protocol

 A word is missing from the sentence “The secondary outcome health professionals…” suggestion: “The secondary outcomes comprise health professionals involved, and the functional outcome reported.” Are there two or three outcomes – later in the protocol it refers to three objectives. Please check this sentence. It may be clearer to write “The second and third objectives comprise health professionals involved and the functional outcome.”

 Eligibility criteria

 Check if “between” is the correct word for the sentence This scoping review will consider the studies investigating family-based interventions to prevent or treat gaming disorders between parents of children and young people, primary caregivers, and professionals (psychiatrists, nursing, occupational therapists, and schoolteachers). “among” may be a better word.

 Context

 Were online/internet-delivered interventions considered? If not, I suggest make it clear if they were excluded.

 Types of evidence sources.

 If grey literature will be excluded, I suggest making this clear. E.g., “This scoping review will consider all peer-reviewed interventional studies, such as…” Then add “grey literature” to the sentence that lists the exclusion criteria.

 I suggest please justify why the search will involve articles published from 2010. Yes, 12 years is an extensive range, however, internet gaming took off in the '90s; why limit it to 2010-22? This is not a problem, but the decision should be justified.

 Discussion

 A further limitation will be articles published before 2010 will not be included.

 I hope these comments are helpful, and best of luck with this interesting and relevant review.

Is the study design appropriate for the research question?

Yes

Is the rationale for, and objectives of, the study clearly described?

Yes

Are sufficient details of the methods provided to allow replication by others?

Yes

Are the datasets clearly presented in a useable and accessible format?

Yes

Reviewer Expertise:

Problem gambling, cognitive behavior therapy, literature reviews

I confirm that I have read this submission and believe that I have an appropriate level of expertise to confirm that it is of an acceptable scientific standard.
